# Long Term Cyclic Pamidronate Reduces Bone Growth by Inhibiting Osteoclast Mediated Cartilage-to-Bone Turnover in the Mouse

**DOI:** 10.2174/1874325000802010121

**Published:** 2008-07-14

**Authors:** K.D Evans, L.E Sheppard, D.I Grossman, S.H Rao, R.B Martin, A.M Oberbauer

**Affiliations:** 1 Department of Animal Science, University of California, Davis, One Shields Avenue, Davis, CA 95616, USA; 2 Ellison Musculoskeletal Research Center, UC Davis Medical Center 4635 Second Avenue, Sacramento, CA 95817, USA

**Keywords:** Pamidronate, growth plate, bisphosphonates.

## Abstract

Bisphosphonates, used to treat diseases exhibiting increased osteoclast activity, reduce longitudinal bone growth through an as yet undefined mechanism. Pamidronate, an aminobisphosphonate, was given weekly to mice at 0, 1.25, or 2.50 mg/kg/wk beginning at 4 weeks of age. At 12 weeks of age, humeral length, growth plate area, regional chondrocyte cell numbers, chondrocyte apoptosis, TRAP stained osteoclast number, and osteoclast function assessed by cathepsin K immunohistochemistry were quantified. Humeral length was decreased in pamidronate treated mice compared to vehicle control mice, and correlated with greater growth plate areas reflecting greater proliferative and hypertrophic chondrocyte cell numbers with fewer hypertrophic cells undergoing apoptosis. Pamidronate treatment increased TRAP stained osteoclast numbers yet decreased cathepsin K indicating that pamidronate repressed osteoclast maturation and function. The data suggest that long term cyclic pamidronate treatment impairs bone growth by inhibition of osteoclast maturation thereby reducing cartilage-to-bone turnover within the growth plate.

## INTRODUCTION

One preferred treatment for human diseases characterized by increased osteoclast activity, such as osteoporosis and osteogenesis imperfecta, is bisphosphonate therapy [1]. Bisphosphonates primarily affect bone remodeling through inhibition of the osteoclast by reducing osteoclast formation and chemotaxis, or increasing osteoclast apoptosis [2-4], although increased osteoblast growth factor expression and lowered articular chondrocyte apoptosis also have been documented [5]. While inhibition of bone resorption is desirable for treating some disease, bone resorption is critical for bone development and skeletal maintenance. Normal bone elongation requires cartilage-to-bone conversion at endochondral growth plates. Treatment with bisphosphonates during the growth period could inhibit the normal osteoclast and chondroclast function necessary for that cartilage-to-bone turnover, thereby impairing linear bone growth. Studies employing bisphosphonates have reported decreased bone formation rate and increased accumulation of mineralized growth plate tissue in the metaphyseal secondary spongiosa [6]. In addition, abnormal bone remodeling and accumulation of mineralized cartilage remnants have been reported for individuals treated with bisphosphonates during growth [7-9].

Very little is known about the effects of bisphosphonates on cells within the active growth plate and whether prolonged inhibition of osteoclasts adversely affects bone growth when bisphosphonates are used during the growth phase on into adulthood. In this study we evaluated the effect of long term cyclic pamidronate treatment, a clinically relevant bisphosphonate, on bone elongation parameters including length, growth plate area, chondrocyte apoptosis, osteoclast numbers, and osteoclast function in a mouse model in an effort to understand how bisphosphonate inhibition of osteoclasts influences bone elongation at the growth plate.

## MATERIALS AND METHODOLOGY

Wild type mice were produced by breeding heterozygous B6C3Fe-a/a-Cola2oim/+ hybrid oim/wt obtained from Jackson Laboratories (Bar Harbor, ME). All mice were housed at constant temperature (70 ± 2°F), fed Purina mouse diet formula 5008 (Purina, St. Louis, MO) and provided tap water ad lib. Wild type mice used in this study were genotypically confirmed [10], weaned at 3 wks of age, and housed according to sex. At 4 wks of age, mice were assigned randomly to experimental groups based on sex (male and female) and pamidronate dose (vehicle control (0 mg/kg/wk), low (1.25 mg/kg/wk), and high (2.50 mg/kg/wk)) with 9 to 10 animals per group. Doses were selected to bracket pamidronate doses therapeutically administered to children [11-13] and that used in previous mouse studies [14]. Beginning at 4 wks of age (±2 days), mice were given weekly intraperitoneal injections of pamidronate (Aredia^TM^, Novartis Pharmaceuticals, East Hanover, NJ) reconstituted in phosphate-buffered saline (PBS) or PBS vehicle alone. At 12 wks of age, mice were killed by CO_2_ narcosis and their humeral bones were collected. All animal procedures were approved by the University of California Davis Institutional Animal Care and Use Committee.

Bilateral humeri were dissected and length measured from the proximal end of the humeral head to the distal aspect of the humeral medial epicondyle. Bones were prepared for histology as previously described [7] except that decalcification was done in 0.5 M EDTA for at least 3 wks [15] after which the bones were individually rinsed for 72 h under running water and stored in 70% ethanol for a minimum of 24 h. Mid-saggital longitudinal sections (6 µm) in the transverse plane were taken at approximately the same depth to permit direct comparisons between bones. Sections were stained with hematoxylin and eosin, and growth plate area measured as the area encompassing the entire growth plate from germinal cell layer to the last intact transverse cartilage septa of the hypertrophic region. Growth plate height was taken from the germinal layer to the last intact transverse cartilage septa of the hypertrophic chondrocyte layer at three locations (medial, mid, and lateral aspects) within the growth plate.

The effect of pamidronate on cell turnover was assessed by TUNEL (Terminal-transferase dUTP Nick End labeling) staining for apoptotic chondrocytes (DeadEnd^TM^ Colorimetric system for tissue sections, Promega, Madison WI). Left humeral transverse sections were taken from five animals for each pamidronate dose. Each slide contained three consecutive slices of bone tissue from the mid-sagittal location of the proximal physis of the humerus again maintaining a constant depth of sectioning to allow for direct comparisons. Sections were counterstained with 0.1% methyl green. Apoptotic cells were identified as having a dark brown staining nucleus and no staining of apoptotic cells was detected in the negative control slides. On each slide, apoptotic cells were counted for each of the three consecutive tissue sections. Normal non-staining cells also were counted to obtain an overview of the relative proportion of apoptosis within the growth plate for each pamidronate dose. Additionally, proportional apoptotic cell counts were taken within the proliferative and hypertrophic regions of the growth plate region of each tissue section in order to assess regional differences in chondrocyte apoptosis. For all growth plate parameters measured, males and females were not significantly different, therefore, females were used for TUNEL analysis and males were used for cathepsin K immunostaining.

Left humeral transverse sections from three mice for each sex by pamidronate dose were rehydrated, then immersed overnight in a reactivation buffer (0.07 M Tris, 0.06 M hydrochloric acid, pH 9.0) and TRAP (tartrate resistant acid phosphatase) stained according to previous studies [16]. Following TRAP staining, slides were rinsed and counterstained with 1% methyl green. TRAP positive osteoclasts were counted to quantify the number of osteoclasts within the metaphyseal region directly below the growth plate on three consecutive bone sections. Total osteoclast counts were taken using an ocular eyepiece grid at a magnification of 400X; the entire grid area (0.0004 mm2) was used and included the total area at the chondro-osseous junction of the growth plate and extended 0.02 mm distally and 0.02 mm medial-laterally. In addition, surface area of calcified cartilage and mineralized bone were taken within the same grid area to account for osteoclast number per surface area and to correlate differences in osteoclast number with potential changes in surface area due to pamidronate dose effect.

Osteoclast function was assessed by cathepsin K staining. Left humeral transverse sections each containing three consecutive slices of bone tissue from the mid-sagittal section of the humeral bone from three males of each pamidronate dose were immunostained for cathepsin K using published methodologies [16]. Briefly, each tissue section was deparaffinized and rehydrated in PBS. Pretreatments included antigen retrieval in a 10 mM citrate buffer for 20 min at 55-60°C. The cathepsin K primary antibody (Santa Cruz Biotechnology Inc., Santa Cruz, CA) was used at a 1:100 dilution per sample. Antibody detection was done according to manufacturer’s directions (Vectastain ABC kit, Vector Laboratories, Burlingame, CA). Negative control slides were prepared by excluding the primary antibody but retaining all the other steps. Internal positive controls were achieved through cathepsin K staining of terminal hypertrophic cells [17]. Sections were counterstained with 0.1% methyl green.

The effects of pamidronate treatment on humeral length, growth plate area, and height were analyzed using least square means analysis of variance that included sex and dose treated as fixed effects (PROC GLM, SAS version 6.07; SAS Institute Inc., Cary, NC). Sex effects were only significant (p < 0.05) for bone length. Within an individual, bone lengths were not significantly different between the right and left sides (p > 0.2) so right and left bone length values were averaged for each animal. Sex effects were not significant (p > 0.2) for all growth plate measurements and cellular evaluation, therefore data from the two sexes were pooled. For all analyses, significance was defined as p < 0.05. Post hoc analyses were done using a Bonferroni adjustment.

Apoptotic cell proportions for the overall growth plate and its regions were analyzed using least square means analysis of variance (PROC MIXED, SAS) with replicate sections for each animal treated as nested values within each pamidronate dose. The effects of pamidronate treatment on number of TRAP stained osteoclast cells and calcified cartilage and bone surface area per grid were analyzed using least square means analysis of variance with dose and sex treated as fixed effects (PROC MIXED, SAS) and replicate sections for each animal treated as nested values within each sex and dose. For TRAP analysis, both sexes were combined for analysis because sex effects did not differ statistically (p > 0.2).

## RESULTS

For humeral bone length, sex was significant (p < 0.01) with males having a longer humerus than females. Bone length was diminished 3% with pamidronate treatment for both males and females (p < 0.05) compared to vehicle treated control humeri (Table **1**). Growth plate area and height were affected by pamidronate dose while sex (p > 0.2) was not significant therefore sexes were combined for further growth plate dimensional analysis. Pamidronate treatment was associated with a larger growth plate area: the high dose treated mice had a 20% increase (p < 0.005) when compared to vehicle treated control mice and the low dose increase was intermediate at 10% (Table** 1**). The enlarged growth plate area was predominantly due to greater growth plate height in pamidronate treated mice: a 57% and 68% increase for low and high doses, respectively, compared to vehicle treated mice.

Growth plate chondrocyte apoptosis and zone cell numbers: The shorter humeral length and greater growth plate area and height observed with pamidronate treatment were associated with a dose-dependent increase in total number of growth plate chondrocytes and a lower proportion of total apoptotic growth plate chondrocytes (Table **1**). The low pamidronate dose did not significantly change total proliferative cell numbers whereas proliferative chondrocyte number was increased with the high dose of pamidronate. Hypertrophic chondrocyte numbers displayed a dose-dependent increase with the highest pamidronate dose increasing hypertrophic cells by 23% relative to vehicle control treated (p < 0.03). The total percentage of apoptotic chondrocytes within the growth plate was significantly lower (~50% decrease) with pamidronate treatment (p < 0.001).

**Fig. (1):**
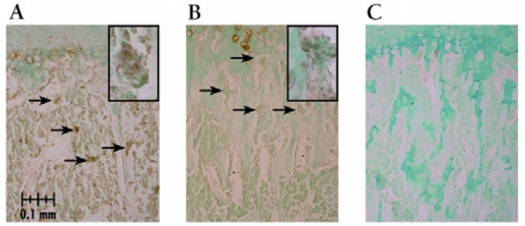
Cathepsin K immunostaining of osteoclasts in growth plate sections from pamidronate treated mice. All sections demonstrated staining in hypertrophic cells which served as an internal positive control. Arrows depict stained osteoclasts lining the trabecular bone in the distal aspect of the proximal metaphysis below the chondro-osseous junction, though not all positively stained osteoclasts are indicated. Magnification is 200X unless noted. **(A)** vehicle control dose treated male with cathepsin K stained osteoclasts, inset photo illustrates an osteoclast stained positive for cathepsin K (400X); **(B)** high pamidronate dose male lacking cathepsin K staining, inset photo (400X) illustrates the lack of cathepsin K staining in osteoclasts at the distal aspect of the proximal metaphysis relative to that depicted in **(A)** and **(C)** negative control slide.

The number of TRAP stained osteoclasts expressed as number per unit of bone area was increased with both pamidronate doses compared to vehicle treated control mice. Greater osteoclast numbers were seen primarily within the metaphysic upon whole bone microscopic observation. Osteoclast numbers were higher with pamidronate treatment although immunohistochemical staining with cathepsin K indicated the activity of those osteoclasts was diminished (Fig. **1**). Osteoclast staining for cathepsin K was virtually absent in the high dose pamidronate treated mice compared to vehicle treated control mice. To verify that greater osteoclast numbers were not due simply to changes in mineralized surface area available, surface areas were quantified. There were no significant differences in the measured metaphyseal mineralized surface area for sex (p > 0.4) or dose (p > 0.1) (data not shown).

## DISCUSSION

Bisphosphonates, particularly pamidronate, are used to increase bone mineral density, decrease fractures and bone pain and thus improve the quality of life for patients with elevated bone resorption such as seen in many osteogenesis imperfecta patients as well as postmenopausal osteoporosis. Improved mechanical properties and decreased bone pain with cyclic pamidronate dosing confirms the role of long term cyclic pamidronate as a palliative treatment [18], yet the effect of bisphosphonate inhibition of osteoclasts on growth plate function is not well defined. In the present study, humoral length was decreased in response to pamidronate, an expected response since alendronate, another bisphosphonate used in clinical trials for osteogenesis imperfecta, showed similar changes on bone length [7]. While alendronate is considered more potent than pamidronate [1], both bisphosphonates elicited similar changes to bone growth indicating both bisphosphonates have comparable biological effects on the growth plate.

While bisphosphonate doses cannot be directly compared between mice and children, correlating human height curves at the 50th percentile suggests that each week of pamidronate treatment in these mice was roughly equivalent to 0.5-1 year of a child receiving treatment during the growth phase [19]. Dosing schemes for children treated with intravenous pamidronate have ranged from 8 mg/kg/yr to 18 mg/kg/yr [11-12] with higher pamidronate doses (114 mg/kg/yr) inducing osteopetrosis [13]. In the present study, the treatment period encompassed roughly 60% of the mouse humerus growth phase [20] and thus, the weekly doses used in this study were lower than the yearly dosages used in children yet still impaired bone elongation in the mice.

Chondrocyte cell turnover from resting to proliferative to hypertrophic and extracellular matrix deposition at endochondral growth plates accounts for the majority of linear bone growth. Treatment with pamidronate inhibited apoptosis throughout the growth plate indicating that the bisphosphonate either directly disrupts normal cellular progression within the growth plate or does so indirectly through osteoclast inhibition resulting in reduced turnover at the chondro-osseous junction. Hypertrophic chondrocytes must undergo apoptosis for normal bone elongation; this apoptosis occurs either by pre-programming or by induction by factors accompanying the invading metaphyseal vasculature [21, 22]. Although bisphosphonates target bone in general, they do concentrate in the metaphysis with very dense uptake in the region of mineralized cartilage just below the growth plate [23]. This would physically position the compound to affect hypertrophic and osteoclast cells, both critical to turnover at the chondro-osseus junction.

In bone, bisphosphonates have a direct inhibitory effect on osteoclast function and morphology [4, 24]. The increased osteoclast numbers with reduced functional activity at the growth plate corroborates those findings. Reduction of osteoclast mediated metaphyseal vessel invasion would restrict the delivery of pro-apoptotic signals to the hypertrophic layer decreasing hypertrophic cell turnover. Mature osteoclasts capable of resorption secrete cathepsin K into the resorption lacuna [25]. Reduced cathepsin K staining indicates an arrest of osteoclast initiation of resorption. Thus, the increase in osteoclasts observed in the present study in response to pamidronate treatment may have been due to 1) decreased osteoclast apoptosis or 2) an increase in osteoclast numbers needed to sustain the basic multicellular unit (BMU) equilibrium [26] but that fail to achieve full osteoclastic maturation and resorptive capacity. Previous studies [2] report an increase in apoptosis for osteoclasts exposed to bisphosphonates suggesting that the increased osteoclast numbers observed in the present study were due to increased recruitment of osteoclast formation with inhibition of their full maturation.

Bisphosphonates also inhibit collagenase-3 expression, an enzyme essential for endochondral bone development [27]. Many studies demonstrate a direct role for collagen degradation in activation of osteoclasts and any reduction in collagenase-3 would result in impaired bone resorption (reviewed in [27]) contributing to the changes observed in the present study. Further, bisphosphonates could have reduced the action of chondroclasts, resorptive cells active at the zone of vascular invasion [28]. Although chondroclasts and osteoclasts were not individually counted, the number of TRAP positive cells was determined from the last intact hypertrophic cell into the primary spongiosa. Thus, any TRAP positive chondroclasts also would have been included in the present data. It is likely that pamidronate would reduce the action of chondroclasts in a manner similar to that proposed for the osteoclast.

The action of bisphosphonates on bone is thought to reflect its action on osteoclasts but other cell types (osteoblasts, tumor cells, monocytes) are known to respond to bisphosphonates [29]. Inhibition of bone growth by bisphosphonates likely reflects an action on multiple cell types. Aminobisphosphonates can inhibit the prenylation of GTP associated proteins Ras, Rac, and Rho [24, 30], factors vital to the signaling pathways that regulate osteoclast, osteoblast, and chondrocyte function. The GTP associated proteins, Rac1 and Rho, antagonistically regulate chondrocyte proliferation, hypertrophy, and apoptosis in much the same manner as they antagonistically act upon osteoclast formation, function, and motility [31]. Thus, in addition to the well-known osteoclast effects of bisphosphonates there may also be a direct effect on the chondrocytes within the growth plate. In this study, it is possible that the increase in proliferative and hypertrophic chondrocyte numbers seen with pamidronate treatment could be due to direct effects of pamidronate on signaling of GTP associated proteins involved in chondrocyte proliferation and or direct inhibitory effects on apoptosis signaling of chondrocytes due to the chondroprotective effects of bisphosphonates [32].

## CONCLUSIONS

This study showed that pamidronate in low and high doses over the majority of the growth phase reduced long bone growth through alterations in growth plate dimensions, chondrocyte apoptosis, and growth plate chondrocyte numbers in conjunction with inhibition of multinucleated osteoclast activity. The bisphosphonate inhibited growth by decreasing chondrocyte turnover *via *inhibition of apoptosis at the hypertrophic layer along with bisphosphonate inhibition of multinucleated osteoclast activity. The inhibition of osteoclast activity may have reduced vessel invasion at the chondro-osseous junction thereby exacerbating the growth inhibitory effect of pamidronate by inhibiting invading vascular apoptotic signals. While this study demonstrated direct effects of bisphosphonates on growth plate function, the beneficial effects of increased bone density and lowered fracture incidence in particular clinical conditions outweigh reduced growth plate activity.

## Figures and Tables

**Table 1.** Pamidronate Effects on Humerus Length, Growth Plate (G.P.) Measurements, and Osteoclast Numbers in Mice

	Control	Low	High	SEM	p Value
**Female Humerus Length, mm**	${\underset{\left(10\right)}{12.29}}^{a}$	${\underset{\left(10\right)}{11.92}}^{b}$	${\underset{\left(10\right)}{11.97}}^{b}$	0.09	0.03
**Male Humerus Length, mm**	${\underset{\left(10\right)}{12.56}}^{a}$	${\underset{\left(10\right)}{12.15}}^{b}$	${\underset{\left(10\right)}{12.12}}^{b}$	0.08	0.009
**G. P. Area, mm^2^**	${\underset{\left(10\right)}{0.2663}}^{a}$	${\underset{\left(10\right)}{0.3067}}^{\mathrm{ab}}$	${\underset{\left(10\right)}{0.3588}}^{b}$	0.019	0.003
**G. P. Height, mm**	${\underset{\left(10\right)}{0.0120}}^{a}$	${\underset{\left(10\right)}{0.0188}}^{b}$	${\underset{\left(10\right)}{0.0197}}^{b}$	0.001	0.001
**TRAP Osteoclasts Per mm^2^ bone**	${\underset{\left(5\right)}{3}}^{a}$	${\underset{\left(5\right)}{6}}^{b}$	${\underset{\left(5\right)}{6}}^{b}$	0.6	0.01
**Proliferative Cell Total**	${\underset{\left(5\right)}{336}}^{a}$	${\underset{\left(5\right)}{359}}^{a}$	${\underset{\left(5\right)}{421}}^{b}$	10	0.001
**Hypertrophic Cell Total**	${\underset{\left(5\right)}{235}}^{a}$	${\underset{\left(5\right)}{276}}^{\mathrm{ab}}$	${\underset{\left(5\right)}{290}}^{b}$	15	0.03
**G.P. Cells Total**	${\underset{\left(5\right)}{658}}^{a}$	${\underset{\left(5\right)}{729}}^{\mathrm{ab}}$	${\underset{\left(5\right)}{804}}^{b}$	17	0.001
**Proliferative Apoptotsis (%)**	$\underset{\left(5\right)}{1.7}$	$\underset{\left(5\right)}{1.9}$	$\underset{\left(5\right)}{1.9}$	0.3	0.60
**Hypertrophic Apoptosis (%)**	${\underset{\left(5\right)}{11.8}}^{a}$	${\underset{\left(5\right)}{4.2}}^{b}$	${\underset{\left(5\right)}{2.5}}^{c}$	0.5	0.03
**Total G. P. Apoptosis (%)**	${\underset{\left(5\right)}{5.7}}^{a}$	${\underset{\left(5\right)}{3.4}}^{b}$	${\underset{\left(5\right)}{2.6}}^{b}$	0.3	0.001

Dose of pamidronate (vehicle control (0 mg/kg/wk), low (1.25mg/kg/wk), or high (2.50 mg/kg/wk) given for 8 wks and evaluated at 12 wks of age. Values are expressed as least square means ± standard error of the means, number of animals (n) is listed below means. Means having different superscripts within each measured parameter are significantly different.
